# Effect of dietary salt intake on epithelial Na^+^ channels (ENaCs) in the hypothalamus of Dahl salt‐sensitive rats

**DOI:** 10.14814/phy2.13838

**Published:** 2018-08-28

**Authors:** Natalie J. Mills, Kaustubh Sharma, Katie Huang, Ryoichi Teruyama

**Affiliations:** ^1^ Department of Biological Sciences Louisiana State University Baton Rouge Louisiana

**Keywords:** Mineralocorticoid receptor, supraoptic nucleus, vasopressin

## Abstract

All three epithelial Na^+^ channel (ENaC) subunits (*α*,* β*, and *γ*) and the mineralocorticoid receptor (MR), a known regulator of ENaC, are located in vasopressin (VP) synthesizing magnocellular neurons in the hypothalamic supraoptic (SON) and paraventricular (PVN) nuclei. Our previous study showed that ENaC mediates a Na^+^ leak current that affects the steady‐state membrane potential of VP neurons. This study was conducted in Dahl salt‐sensitive (Dahl‐SS) rats to determine if any abnormal responses in the expression of ENaC subunits and MR occur in the hypothalamus and kidney in response to a high dietary salt intake. After 21 days of high salt consumption, Dahl‐SS rat resulted in a significant increase in *γ* ENaC expression and exhibited proteolytic cleavage of this subunit compared to Sprague–Dawley (SD) rats. Additionally, Dahl‐SS rats had dense somato‐dendritic *γ* ENaC immunoreactivity in VP neurons, which was absent in SD rats. In contrast, SD rats fed a high salt diet had significantly decreased *α* ENaC subunit expression in the kidney and MR expression in the hypothalamus. Plasma osmolality measured daily for 22 days demonstrated that Dahl‐SS rats fed a high salt diet had a steady increase in plasma osmolality, whereas SD rats had an initial increase that decreased to baseline levels. Findings from this study demonstrate that Dahl‐SS rats lack a compensatory mechanism to down regulate ENaC during high dietary salt consumption, which may contribute to the development of hypertension.

## Introduction

In the paraventricular (PVN) and supraoptic nuclei (SON) of the hypothalamus, magnocellular neurosecretory cells (MNCs) synthesize the neurohypophysial hormones, vasopressin (VP) and oxytocin (OT). Synthesis and release of VP into the general circulation is promoted by physiological demands to maintain body fluid homeostasis. During times of hyperosmolarity (Brimble and Dyball 1977), hypovolemia (Harris et al. 1975), and hypotension (Khanna et al. 1994), VP release is enhanced to produce pressor and antidiuretic effects to counter these physiological changes (Antunes‐Rodrigues et al. 2004). Additionally, VP is released from the soma and dendrites of VP neurons located in the PVN to enhance the activity of the parvocellular neurons that project to the rostral ventrolateral medulla to cause an increase in sympathetic outflow and blood pressure (Son et al. 2013). Given these systemic effects of VP, it is clear that VP plays an essential role in coordinating neuroendocrine and autonomic activities to maintain cardiovascular homeostasis.

The nonvoltage dependent, amiloride‐sensitive, epithelial Na^+^ channel (ENaC) is expressed within the apical membrane of the epithelium involved in transepithelial Na^+^ transport (Canessa et al. 1994; Harris et al. 2008), such as in the distal colon and in the renal collecting duct of the kidney (Kellenberger and Schild 2002). ENaC expression was also demonstrated in various regions of the brain involved in cardiovascular control, such as the PVN, SON, and choroid plexus (Amin et al. 2005). Our previous study showed that all ENaC subunits (*α*,* β*, and *γ*) are expressed specifically in VP MNCs, and functional ENaC channels modulate the membrane potential of VP neurons by mediating a Na^+^ leak current (Teruyama et al. 2012). Additionally, we demonstrated the mineralocorticoid receptor (MR), a known ENaC regulator, is present in VP neurons (Teruyama et al. 2012; Haque et al. 2015).

It is well‐known that dietary salt regulates ENaC expression in aldosterone‐sensitive epithelia (Garty and Palmer 1997; Stokes and Sigmund 1998), such as in the renal collecting duct (Masilamani et al. 1999). Interestingly, central administration of amiloride, an ENaC blocker, and its analog, benzamil, attenuates hypertension in several animal models of hypertension (Gomez‐Sanchez and Gomez‐Sanchez 1994, 1995; Nishimura et al. 1998; Keep et al. 1999). Additionally, we recently demonstrated that ENaC activity in VP neurons is enhanced by increased dietary salt intake in normotensive rats (Sharma et al. 2017). These findings suggest that central ENaC expression and activity are involved in the development of hypertension. The effect of dietary salt on ENaC expression within VP neurons during salt‐sensitive hypertension remains unknown. This study was conducted to examine ENaC expression during high dietary salt intake in salt‐sensitive states utilizing Dahl salt‐sensitive (Dahl‐SS) rats, an established animal model for salt‐sensitive hypertension. Findings from this study indicate that ENaC expression within VP neurons is increased in Dahl‐SS rats during high dietary salt intake, which may provide some insight into the etiology of salt‐sensitive hypertension.

## Materials and Methods

### Animals

Male Sprague–Dawley (SD) and Dahl‐SS rats (ENVIGO, Indianapolis, IN) weighing between 260 and 300 g were used for this study. SD rats from which Dahl‐SS rats were originally derived (Dahl et al. 1962; Rapp 1982) were used as a control strain. Rats were housed on a 12:12 h light–dark cycle. All protocols were approved by the Institutional Animal Care and Use Committees of Louisiana State University.

### Tissue sample for Real‐time RT‐PCR and Semiquatitative immunoblotting

Rats were fed standard chow (0.4% NaCl) or high salt (8% NaCl) (Teklad diet Indianapolis, IN) for 21 days and water ad libitum. On the 21^st^ day, animals were sacrificed and tissue was collected for real‐time RT‐PCR or semiquatitative immunoblotting. Rats were deeply anaesthetized using Ketamine‐Xylazine (9:1, 100 mg/kg i.p.) and approximately 1 ml of blood was collected from each anesthetized animal by a 21‐gauge needle inserted into the left ventricle directly before cardiac perfusion. Animals were then perfused through the heart with cold artificial cerebral spinal fluid (aCSF), in which NaCl was replaced by equiosmolar amount of sucrose containing (in mM): 210 Sucrose, 3 KCl, 2.0 CaCl_2_, 1.3 MgCl_2_, 1.24 NaH_2_PO_4_, 25 NaHCO_3_, 0.2 ascorbic acid, and 10 D‐glucose (pH 7.4). The brain of each rat was removed and three coronal slices (500 *μ*m) were collected using a vibrating microtome (Leica VT1200; Leica, Mannheim, Germany). The brain slices were trimmed to the smallest possible size that contained the entirety of the SON and PVN. Other brain areas that are known to express ENaC, namely the vascular organ of lamina terminalis (OVLT) (Miller and Loewy 2013), area postrema (Miller et al. 2013), and subfornical organ (SFO) (Wang et al. 2016), were not included. The trimmed hypothalamic slices were then stored in *RNAlater* (Qiagen, Valencia, CA) at −20°C for real‐time RT‐PCR or RIPA Buffer (Sigma‐Aldrich, St Louis, MO) for semiquatitative immunoblotting. The PVN and SON regions were analyzed together to increase the ability of detecting changes in target gene expression and abundance. Additionally, during tissue collection for real‐time RT‐PCR, one kidney from each animal was also removed, minced, and saved in RNA*later* for processing.

### Real‐time RT‐PCR

Total RNA was isolated using TriReagent (Sigma‐Aldrich, St Louis, MO) according to the manufacture's instructions and tissue was homogenized using a tissue lyser (Qiagen, CA). Lysate was then treated with chloroform (Thermo‐Fisher Scientific) and centrifuged at 15,294 × *g* for 15 min. Total RNA was precipitated from the aqueous phase using isopropyl alcohol and washed with ethanol. The precipitate was suspended in 20 *μ*l RNAase‐free water (Qiagen, CA). The concentration and quality of the isolated RNA was assessed using a spectrophotometer (NanoDrop 1000, ThermoFisher Scientific, Waltham, MA). Isolated mRNA was reverse transcribed to cDNA using oligo dT and M‐MLV reverse transcriptase (Sigma‐Aldrich, St. Luis, MO) and used in real‐time RT‐PCR analysis. ABI ViiA‐7 sequence detection system (ABI Applied Biosystem, Grand Island, NY) was used in conjunction with SYBR Select Master Mix (ABI Applied Biosystem, Grand Island, NY). All samples were measured with three replicates and the two closest cycle threshold (Ct) values were averaged for each sample. Quantitative PCR primers were designed using Primer 3 software (Whitehead Institute for Biomedical Research) and purchased from Sigma‐Aldrich (St. Louis, MO). All primer sequences were specifically designed to amplify the cDNA of interest (Table 1) and were previously used successfully (Amin et al. 2005; Teruyama et al. 2012). The effect of the treatments was examined through the relative change in the specific target genes, which were calculated through the comparative cycle threshold method. For each sample, the average cycle threshold (Ct) value of the target gene was subtracted by the housekeeping (Cyclophilin B) Ct value to obtain the ΔCt value. The ΔCt values of the designated control group were averaged and subtracted from each sample to generate the ΔΔCt value. The relative change was calculated for each sample by using the formula 2 ^− ΔΔCt^. All results are shown as relative change in comparison to the control. The Student's *t* test was used for comparison between treatment groups. Differences were considered to be statistically significant at *P* < 0.05.

**Table 1:** Primer sequences used to detect genes of interest

Gene	Primer Sequence	Amplicon Size, bp
*α*ENaC Forward Reverse	5′‐CCCAAGGGAGTTGAGTTCTG‐3′ 5′‐AGGCGCCCTGCAGTTTAT‐3′	76
*β*ENaC Forward Reverse	5′‐GGACCAGAGCTAAATATCACC‐3′ 5′‐CGGTAGTTGAACTCTTGGAAGTAGA‐3′	78
*γ*ENaC Forward Reverse	5′‐CCAGTACAGCCAGCCTCTG‐3′ 5′‐CTGGTACAACTGGTAGTAGCAATACAT‐3′	91
MR Forward Reverse	5′‐CTCCCTAACATGTCCTAGAAAAGC‐3′ 5′‐AGAACGCTCCAAGGTCTGAG‐3	112
OT Forward Reverse	5′‐CGCCTGCTACATCCAGAACT‐3′ 5′‐CCGCAGGGAAGACACTTG	81
VP Forward Reverse	5′‐TCCGACATGGAGCTGAGAC‐3′ 5′ –GGGCAGGTAGTTCTCCTCCT‐3′	147

### Plasma osmolality

Collected blood samples were centrifuged at ~15,000 *g* for 20 min at 4°C and the supernatant was collected and placed in a chilled 1.5 mL centrifuge tube. The collected supernatant was centrifuged for 5 min at 15,294 × *g* at 4°C, and the resulting supernatant was collected and stored at −20°C for later processing. Osmolality of the blood samples was measured using an osmometer (5004 Micro‐osmette, Precision System Inc., Natick, MA). A one‐way ANOVA was used for comparison between treatment groups. Differences were considered to be statistically significant at *P* < 0.05.

### Semiquatitative immunoblotting

Total protein lysate samples were collected from the hypothalamic slices. Tissue samples were rinsed with cold phosphate buffer solution and placed in microcentrifuge tubes containing 250 *μ*L of chilled complete lysis buffer that was made fresh before each experiment. Complete buffer contained: RIPA buffer (50 mmol/L NaCl, 1.0% IGEPAL^®^ CA‐630, 0.5% sodium deoxycholate, 0.1% SDS, 50 mmol/L Tris, pH 8.0; Sigma‐Aldrich), protease inhibitors (ProteoGuard™ EDTA‐free protease inhibitor cocktail; Takara Clontech), 1 mmol/L EGTA, 1 mmol/L EDTA, and 1 mmol/L DTT (Sigma‐Aldrich, St. Louis, MO). Samples were briefly homogenized with an electric tissue lyser (Qiagen, Valencia, CA) and allowed to stand on ice for 10 min. Lysed samples were centrifuged for 20 min at 12,000 rpm at 4°C. The pellet was discarded and the supernatant (total lysate) was transferred to a clean tube to be stored at −20°C for later processing.

The protein concentration was determined (BCA kit; Pierce Chemical Co., Rockford, IL); samples (15–20 *μ*g of protein) were denatured by heating in 1x Laemmli buffer (Bio‐Rad, Hercules, CA) for 5 min at 95°C. Samples were resolved in a 4–15% SDS‐PAGE (Mini PROTEAN Precast Gels, Bio‐Rad, Hercules, CA) gradient gel in Tris‐glycine buffer (25 mmol/L, 192 mmol/L glycine, 0.1% SDS, pH 8.3; Bio‐Rad) and transferred to a PVDF membrane (Bio‐Rad, Hercules, CA) under wet conditions (50V for 2 h at 4°C). To determine a successful transfer, the membrane was stained with Ponceau S solution (Sigma‐Aldrich, St. Louis, MO) for approximately 1 min. The membrane was destained with 0.1 mol/L NaOH for 30 sec followed by a 1 min TBST (Tris‐GlycineTween; 50 mmol/L Tris base, 200 mmol/L NaCl, 0.05% Tween 20) wash. The membrane was treated with 5% nonfat milk buffer (NFM; Bio‐Rad) for 1 h at room temp. The membrane was incubated overnight at 4°C with the selected primary antibody dissolved in 5% NFM buffer. A final concentration for all primary antibodies was 1 *μ*g/ml. The membrane was washed three times for 10 min each with TBST and incubated with goat‐anti‐rabbit horseradish conjugated secondary antibody (1:10,000; Bio‐Rad) dissolved in 5% NFM buffer at room temp for 2 h followed by six TBST washes for 10 min each. Protein bands were detected through chemiluminscence reagents (Clarity™ Western ECl Substrate Kit; Bio‐Rad) and imaged using a gel imaging system (ChemiDoc™ XRS+; Bio‐Rad). Densitometric analyses of the images were performed using ImageJ (National Institutes of Health, Bethesda, MD). Measurements were normalized by the load control, GAPDH. Densometric measurements were analyzed as a comparison between rat strain and dietary treatment for individual animals using a one‐way ANOVA and considered significantly different at *P *<* *0.05.

### Immunocytochemistry

Rats fed high salt or control diet for 21 days were deeply anaesthetized using Ketamine‐Xylazine (9:1, 100 mg/kg i.p.) and transcardially perfused with 0.01 mol/L sodium phosphate buffered saline (PBS, pH 7.2) followed by 4% paraformaldehyde in 0.1 mol/L PBS (pH 7.2). Animals were decapitated and the heads were postfixed in the same fixative for 1–3 days. Brains were removed and placed in 20% sucrose in 0.1 mol/L PBS overnight for cryoprotection. Coronal sections at 40 *μ*m were obtained by a sliding microtome (SM2010R; Leica, Mannheim, Germany). Six brain slices containing the SON, collected at every 5th slices, for each animal were incubated with the primary antibody against *γ*ENaC at a dilution of 1:4000 in PBS containing 0.5% Triton X‐100 (PBST) at 4°C with continuous, gentle agitation overnight. The antibodies used for the ENaC subunits were a generous gift from Dr. Mark A. Knepper (National Institutes of Health, Bethesda, MD) and were previously described in great detail (Masilamani et al. 1999). Slices were incubated with biotinylated goat‐anti‐rabbit IgG (1:500 in PBST; Vector, Burlingame, CA) for 2 h followed by incubation with the ABC complex (1:500 in PBST, Vector, Burlingame, CA) for 1 h at room temperature. Finally, slices were incubated with diaminobenzidine (DAB; Vector, Burlingame, CA) for 6 min to visualize immunoreactivity. The brains were mounted on gelatin‐coated slides, dehydrated, cleared, and cover slipped with Permount (Thermo Fisher Scientific, Waltham, MA). Images of the SON were acquired digitally with a microscope equipped with a digital camera (Eclipse 80i with DS‐QiMc, Nikon, Tokyo, Japan). Background optical density measurements were collected for each section and cell optical density measurements were subtracted to obtain normalized values using ImageJ (National Institutes of Health, Bethesda, MD). Normalized measurements that had an optical density 75% higher than the background measurement was classified as having dense immunocytochemistry staining. The number of immunoreactive cells for each animal was obtained from both sides of the SON in all six slices using the Cell Counter plugin in ImageJ (National Institutes of Health, Bethesda, MD). Differences in the number of immunoreactive cells were analyzed using a one‐way ANOVA and considered significantly different at *P *<* *0.05.

### Time course plasma osmolality collection

A separate set of male SD and Dahl‐SS rats were used to measure the changes in plasma osmolality over time in response to dietary salt. Blood samples (0.1 mL) were collected every morning, alternating between left and right tail veins. Samples were stored in 4°C for 30 min to let the blood clot and centrifuged at ~11,000 *g* for 8 min at 4°C. The supernatant was collected to check the osmolality as described above. After the initial 7 days of sampling on control diet to measure the baseline plasma osmolality of each animal, all rats were fed high salt (8% NaCl) diet ad libitum for 22 days with free access to drinking water.

## Results

### Dietary salt increases hypothalamic γENaC, OT, and VP expression and plasma osmolality in Dahl‐SS rats

**Figure 1:**
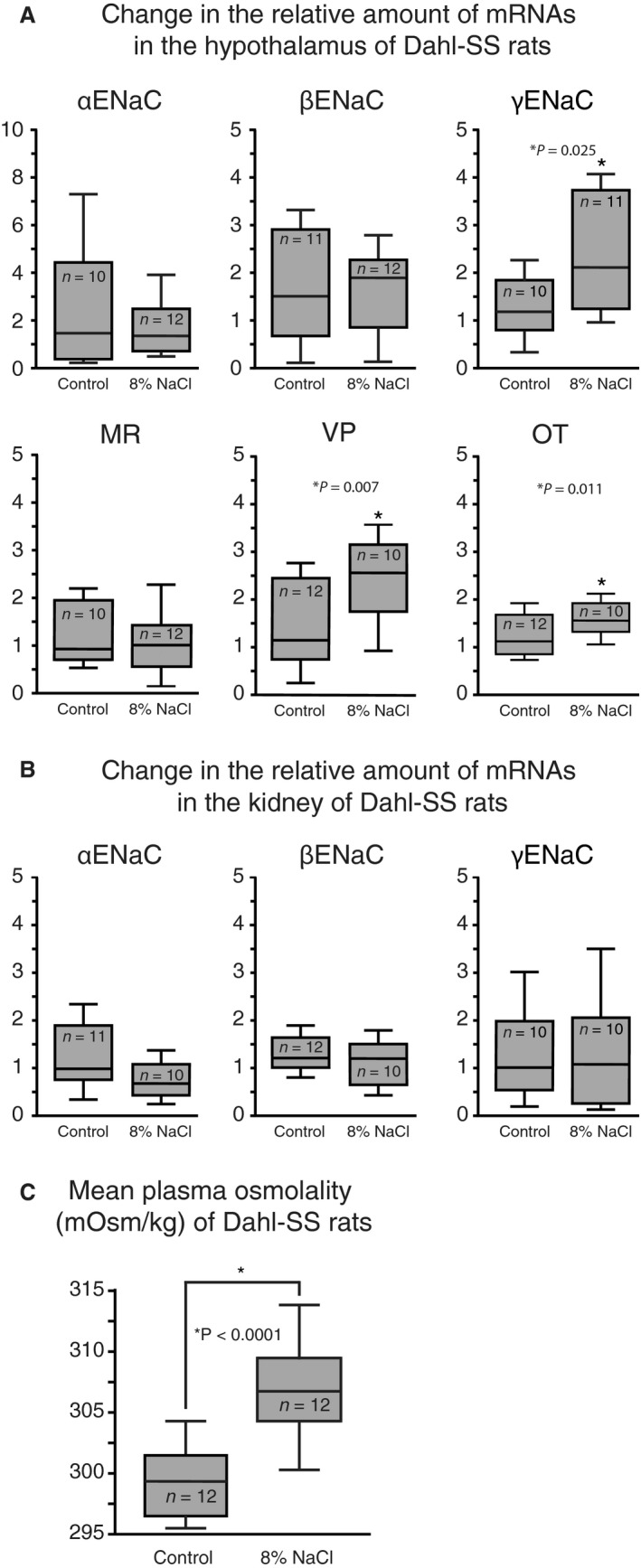
Dietary salt intake increases hypothalamic *γ* ENaC, VP, and OT expression in Dahl‐SS. (A) Dahl‐SS rats fed high salt diet for 21 days had significantly higher *γ* ENaC, VP, and OT mRNA expression in the hypothalamus compared to animals fed control diet, whereas MR,* α*‐ and *β* ENaC hypothalamic mRNA expression was not affected by dietary treatment. (B) No significant difference was detected in ENaC mRNA expression in the kidney between animals fed high salt diet or control. (C) Dahl‐SS fed high salt diet had significantly higher plasma osmolality compared to those fed control diet.

The effect of dietary salt on the expression of ENaC subunits, VP, OT, and MR in the hypothalamus of Dahl‐SS rats was assessed by the relative difference in the amount of mRNA between groups for each of the genes (Fig. 1A). Significantly higher *γ*ENaC subunit expression was observed in Dahl‐SS rats fed a high salt diet compared to those fed the control diet (*P* = 0.025). In contrast, there was no difference in the *α*‐ and *β*ENaC subunit expressions between dietary treatments. Expression of VP and OT was also significantly increased in response to high dietary salt compared to that of the control diet (*P* = 0.007 and *P* = 0.011). Additionally, ENaC expression in the kidney was examined in Dahl‐SS rats fed control or high salt diet. In contrast to hypothalamic ENaC expression, no difference was observed in kidney ENaC subunit expression between treatment groups (Fig. 1B). In addition to tissue collection, plasma samples were collected from rats fed a high salt or a control diet for 21 days (Fig. 3C). Dahl‐SS rats fed the high salt diet exhibited a significantly higher plasma osmolality compared to those fed the control diet (*P* < 0.001).

### SD rats fed high dietary salt have decreased αENaC in the kidney and MR expression in the hypothalamus

**Figure 2:**
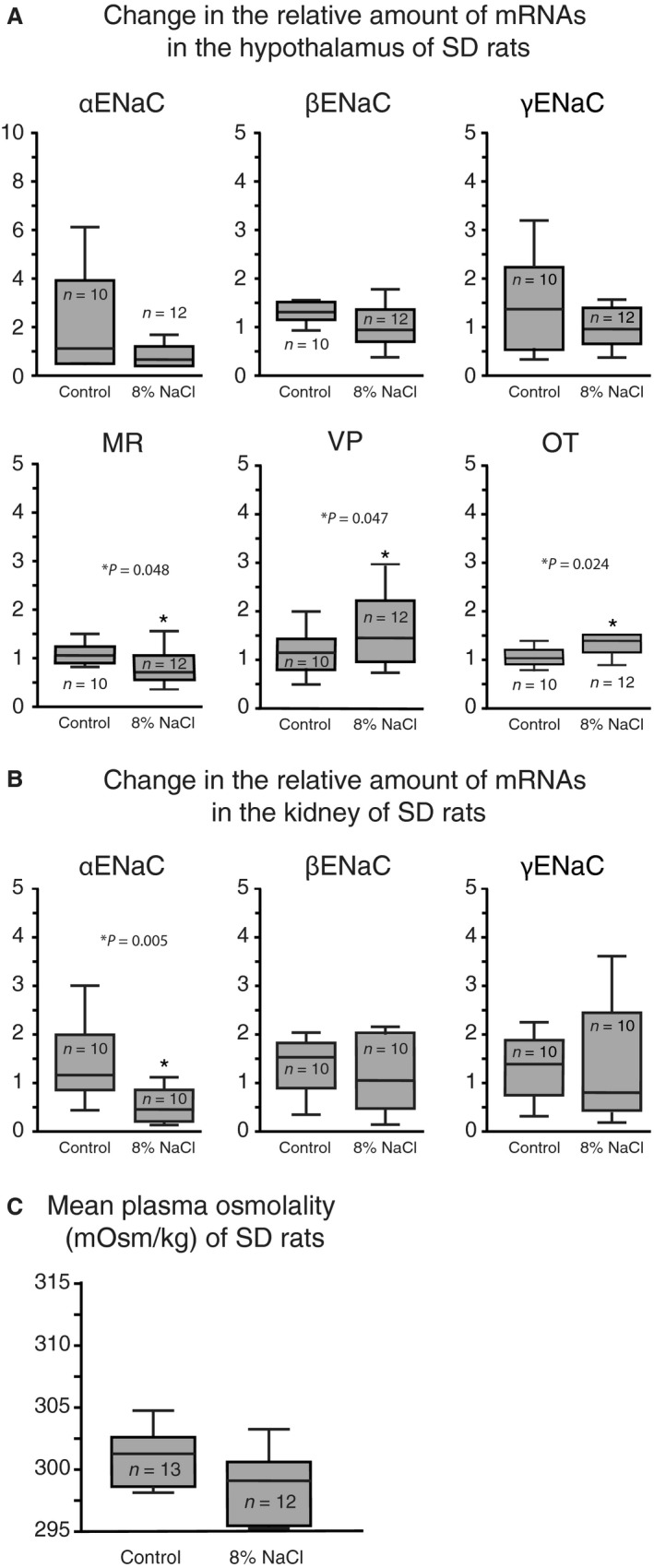
SD rats fed high dietary salt had decreased MR in the hypothalamus and *α* ENaC expressions in the kidney. (A) No significant difference was detected in hypothalamic ENaC subunit expression between treatment groups; however, SD rats fed high salt diet had significantly lower MR mRNA expression and significantly higher VP and OT mRNA expression compared to animals fed control diet. (B) Expression of *α* ENaC mRNA in the kidney was significantly lower in animals fed high salt diet compared to those fed control diet; however, no difference was detected in the *β*‐ and *γ* ENaC expressions between diet treatments. (C) SD rats fed high salt diet for 21 days had no significant difference in plasma osmolality compared to animals fed control diet.

The effect of dietary salt was also assessed on the hypothalamic expression of ENaC subunits, VP, OT, and MR in SD rats (Fig. 2A). No difference was observed in the hypothalamic ENaC subunit expression between dietary treatments. Hypothalamic MR expression, however, was significantly lower in SD rats fed a high salt diet compared to those fed the control diet (*P* = 0.048). Expression of VP and OT was significantly higher in SD rats fed high salt diet (*P* = 0.047 and *P* = 0.024, respectively). Additionally, ENaC expression in the kidney was examined (Fig. 2B), and SD rats fed a high salt diet had significantly lower *α*ENaC expression compared to those fed the control diet (*P* = 0.005). There was no detectable difference in the kidney *β*‐ and *γ*ENaC expressions between dietary treatments. In addition to tissue collection, plasma samples were collected from rats fed a high salt or a control diet for 21 days (Fig. 2C). SD rats showed no difference between dietary treatments. There was no difference detected in the plasma osmolality of Dahl‐SS and SD rats fed the control diet.

### Dahl‐SS rats have increased hypothalamic *γ*ENaC abundance and proteolytic cleavage

**Figure 3:**
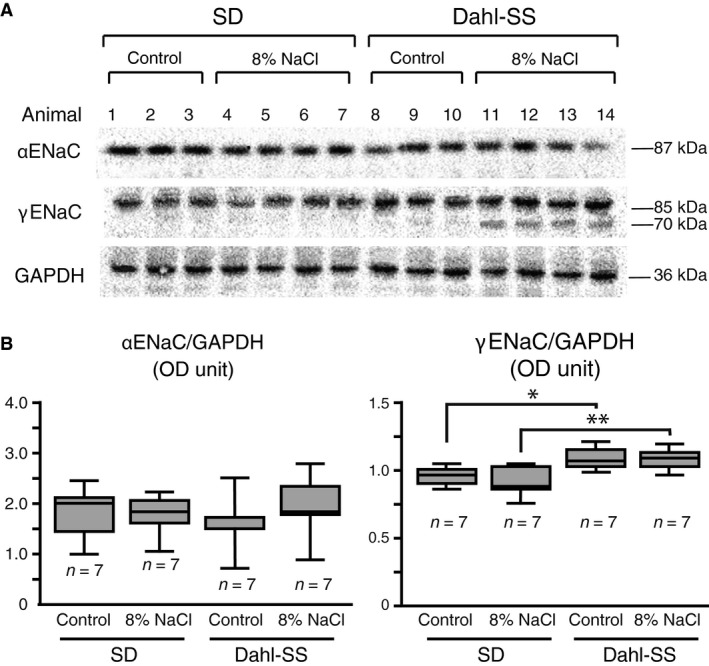
Dahl‐ SS rats have increased *γ* ENaC abundance and dietary salt intake promotes proteolytic cleavage of *γ* ENaC. (A) Immunoblots showing the abundance and molecular weights of ENaC subunits in SD and Dahl‐SS rats fed high salt or control diet for 21 days. For each blot, each lane is representative of an individual animal and loaded with protein extract from the hypothalamus. Antibodies detected a single band for *α* ENaC and *γ* ENaC at approximately 87 kDa and 85 kDa, respectively, in SD rats fed control or high salt diet and Dahl‐SS rats fed control diet. Dahl‐SS rats fed high salt diet displayed a second, smaller band for *γ* ENaC at approximately 70 kDa. No band was detected for *β* ENaC for the extract regardless of strain or diet treatment. (B) Densometric measurements for each band of the *α* ENaC immunoblot detected no difference between strains or dietary treatments; however, densometric measurements for each band of the *γ* ENaC immunoblot demonstrated significantly higher abundance in Dahl‐SS rats fed control diet compared to SD rats fed control diet (*P = 0.017) and Dahl‐SS rats fed high salt diet compared to SD rats fed high salt diet (**P = 0.007).

Proteins were extracted from the hypothalamus of SD and Dahl‐SS rats fed control or high salt diet for 21 days. The effect of dietary salt intake on the ENaC subunits abundance was assessed by semiquantitative immunoblotting (Fig. 3). From the protein extract, a distinct band was detected at the predicted molecular weight of 87 kDa and 85 kDa by the *α*ENaC and *γ*ENaC antibodies, respectively (Fig. 3A). A smaller, second band with a molecular weight of 70 kDa was detected by the *γ*ENaC antibody in Dahl‐SS rats fed the high salt diet. There was no band detected by the *β*ENaC antibody in the protein extract from the hypothalamus. Normalized optical density (OD) measurements were compared between strains and showed that Dahl‐SS rats that were fed the control diet had a significantly higher abundance of the *γ*ENaC subunit (0.883 ± 0.026 OD) compared to SD rats fed the control (0.761 ± 0.029 OD; *P* = 0.046) or high salt (0.7105 ± 0.041 OD; *P* = 0.009) diets. There was no difference in *α*ENaC abundance between strains or diet (Fig. 3B).

### Dahl‐SS rats fed a high salt diet have a prolonged increase in plasma osmolality

**Figure 4:**
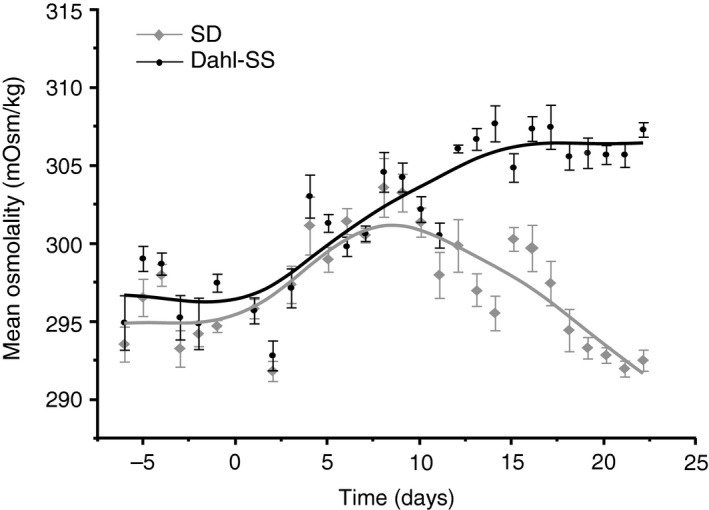
Dahl‐SS rats fed high salt diet had a prolonged increase in plasma osmolality. SD rats that consumed high salt diet for 23 days had an initial increase in plasma osmolality that peaked at day 8 and then gradually returned to baseline measurements by day 21. Dahl‐SS rats had a prolonged increase in plasma osmolality that peaked and plateaued at day 15.

To further examine the plasma osmolality difference observed, a time course collection of plasma osmolality was completed in SD and Dahl‐SS rats fed the high salt diet for 22 days (Fig. 4). Plasma samples were collected for 7 days prior to starting the high salt diet to establish a baseline osmolality for each group, which showed no difference between strains. Within two days of starting the high salt diet, both strains had an increase in plasma osmolality. SD rats had an increase in plasma osmolality until day 8 and then had a steady decrease to baseline measurements by day 19. Dahl‐SS rats had a prolonged increase in plasma osmolality that plateaued at the peak after 15 days on the high salt diet.

### Dahl‐SS rats have increased somato‐dendritic γENaC immunoreactivity in the SON compared to SD rats

**Figure 5:**
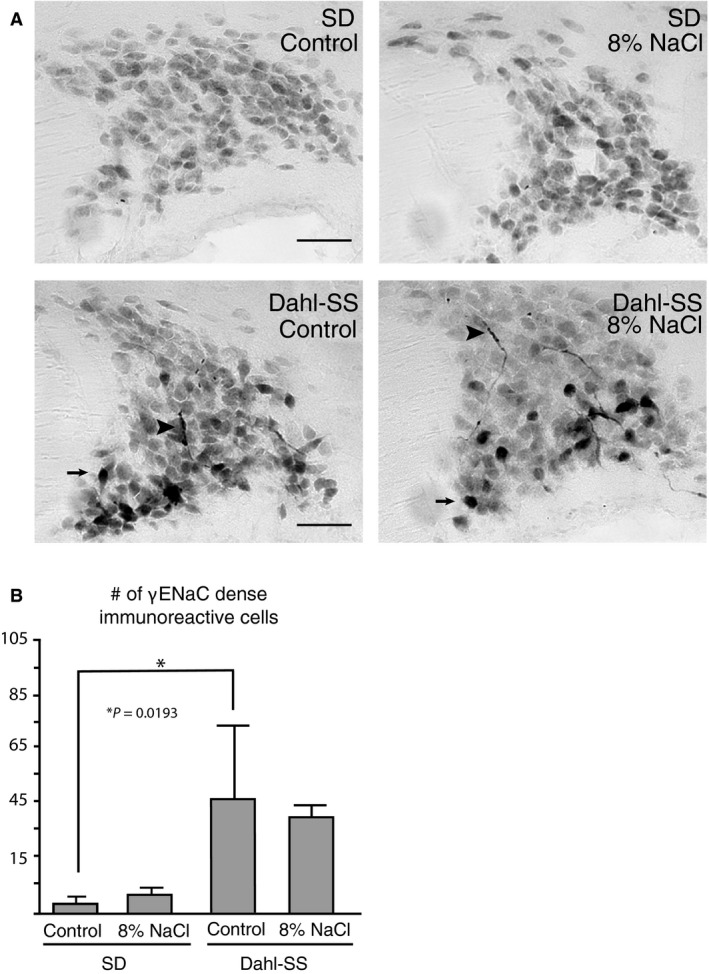
Dahl‐SS rats have increased somato‐dendritic γENaC immunoreactivity in the SON (A) Photomicrographs shown are representative of an animal for each strain and diet treatment. Immunoreactivity to γENaC was found exclusively in the MNCs of the SON in both SD and Dahl‐SS rats regardless of dietary treatments; however, Dahl‐SS rats displayed dense cellular immunoreactivity (arrows) as well as immunoreactive processes (arrowheads), which were not observed in SD rats. (B) The number of dense immunoreactive cells was significantly higher in Dahl‐SS rats compared to SD rats fed control diet; however, no difference was observed between Dahl‐SS rats fed control and high salt diet.

Immunoreactivity of the *γ*ENaC subunit was examined in the SON of SD and Dahl‐SS fed a control or a high salt diet (Fig. 5; *n* = 3). Localization of *γ*ENaC immunoreactivity among all the animals examined was consistent and found exclusively in MNCs in the SON (Fig. 5A). Dahl‐SS rats fed the control diet had significantly more dense‐immunoreactive cells in the SON (46.67 ± 21.7) compared to SD rats (1 ± 1) that were fed the control diet (*P* = 0.019; Fig. 5B). Intra‐strain analysis found no difference in the number of dense‐immunoreactive cells between dietary treatments. In addition to differences in immunoreactivity, Dahl‐SS rats had *γ*ENaC immunoreactive processes present (Fig. 5A). SD rats had no immunoreactive processes detected in either dietary treatment. The total number of *γ*ENaC immunoreactive cells in the SON was not affected by dietary treatment or strain type.

## Discussion

This study demonstrated that the expression and abundance of ENaC within the hypothalamus of Dahl‐SS rats is influenced by dietary salt. Specifically, Dahl‐SS rats fed a high salt diet had increased *γ*ENaC expression in the hypothalamus, whereas *α*ENaC and *β*ENaC subunit expressions remained at basal levels. Regardless of diet treatment, Dahl‐SS rats have higher *γ*ENaC abundance in the hypothalamus compared to normotensive SD rats. In the hypothalamic tissue examined, *γ*ENaC immunoreactivity was located prominently in the VP MNCs of the SON and PVN (Teruyama et al. 2012), suggesting that changes in *γ*ENaC expression in response to dietary salt intake occurred specifically in VP MNCs.

It was previously shown that aldosterone is synthesized in the hypothalamus (MacKenzie et al. 2000; Gomez‐Sanchez and Gomez‐Sanchez 2003; Gomez‐Sanchez et al. 2005) and contributes to the development of salt‐dependent hypertension in Dahl‐SS rats (Huang et al. 2009; Gomez‐Sanchez et al. 2010). Central aldosterone synthesis is higher in Dahl‐SS rats compared to SD rats and inhibition of aldosterone synthase attenuated the salt induced hypertension in Dahl‐SS (Gomez‐Sanchez et al. 2010). A previous study (Huang et al. 2009) also demonstrated that Dahl‐SS rats fed a high salt diet had increased hypothalamic aldosterone levels independent from plasma aldosterone with no change in central MR expression. Furthermore, both aldosterone synthase (Wang et al. 2016) and 11*β*–hydroxysteriod dehydrogenase type 2 (11*β*‐HSD2), enzymes that promote aldosterone binding to MR (Funder JW, 1995; Naray‐Fejes‐Toth et al. 1998) are expressed in MNCs in the SON (Haque et al. 2015). These findings collectively suggest the increased *γ*ENaC expression observed in Dahl‐SS rats fed a high salt diet is a result of increased aldosterone synthesis by VP‐MNCs. Unlike SD rats fed a high salt diet, a decrease in hypothalamic MR expression was not observed in Dahl‐SS rats in this study. Inability of Dahl‐SS rats to downregulate hypothalamic MR expression, therefore, may contribute to the increased *γ*ENaC in VP‐MNCs in addition to the increased synthesis of hypothalamic aldosterone.

Although full activity of ENaC requires all three subunits, changes in specific ENaC subunit expression affects channel activity. Studies completed in cultured collecting duct cells and human lung adenocarcinoma cells showed that selectively increasing *γ*ENaC expression by an adenovirus system resulted in a threefold increase in Na^+^ transport (Volk et al. 2005; Husted et al. 2007) and a longer channel half‐life (Volk et al. 2005). It was demonstrated in *Xenopus laevis* oocytes that the *γ*ENaC subunit has specific domains that are important in increasing channel surface expression and function (Konstas and Korbmacher 2003). These findings, in addition to our previous study examining the effects of dietary salt in SD rats (Sharma et al. 2017), suggest that ENaC activity is increased in VP neurons of Dahl‐SS rats fed high salt diet.

Our study demonstrates that Dahl‐SS rats have a higher hypothalamic *γ*ENaC abundance compared to SD rats regardless of diet treatment. Thus, the abundance of *γ*ENaC in VP MNCs is constitutively higher in Dahl‐SS rats than in SD rats. In addition, the Dahl‐SS rats fed a high salt diet resulted in a shift in the molecular weight of the *γ*ENaC from 85 to 70 kDa. The proteolytic processing of ENaC is important for the regulation of ENaC activity (Rossier 2004; Kleyman et al. 2006). The proteolytic cleavage of the *γ*ENaC subunit releases an extracellular inhibitory domain (Passero et al. 2010), which is necessary for full channel activation (Berman et al. 2015; Shobair et al. 2016) and enhanced activity (Hughey et al. 2004). This shift in molecular weight was documented in the kidney collecting tubule during salt deprivation to prevent sodium loss (Masilamani et al. 1999). The emergence of the 70 kDa proteolytic *γ*ENaC without change in the abundance of 85 kDa noncleaved *γ*ENaC suggests not only an overall increase in *γ*ENaC expression, but also activation of *γ*ENaC in VP MNCs in response to a high dietary salt intake among Dahl‐SS rats.

Plasma aldosterone is an important regulator of ENaC activity within epithelia, and circulating levels are usually inversely correlated with dietary salt intake (Oki et al. 2012; Funder JW, 2017). During the time of sodium restriction, plasma aldosterone levels are elevated and result in increased *α*ENaC expression in the kidney distal tubule to prevent sodium waste (Asher et al. 1996; Escoubet et al. 1997; Stokes and Sigmund 1998; Masilamani et al. 1999; Sharma et al. 2017). Conversely, during salt loading, plasma aldosterone levels decrease (Oki et al. 2012; Funder JW, 2017). Our study shows that SD rats fed a high salt diet for 21 days have decreased kidney *α*ENaC expression compared to SD rats fed the control diet, which may be caused by low plasma aldosterone levels to promote sodium excretion in the urine. In addition to the decreased hypothalamic MR expression, decreased kidney *α*ENaC during high salt diet intake may be part of a compensatory mechanism to maintain sodium homeostasis and prevent development of salt‐induced hypertension. This is further supported by the plasma osmolality measurements that showed SD rats had an initial rise is plasma osmolality during high salt consumption, which peaked on day 8 and then returned to levels comparable with baseline measurements. It should be noted that our previous study detected increased hypothalamic ENaC expression after 7 to 10 days on a high salt diet (Sharma et al. 2017), which appears to correspond to the detected increase in plasma osmolality shown in this study. Changes in hypothalamic ENaC expression were not detected after 21 days on a high salt diet, and plasma osmolality levels were comparable to baseline measurements, suggesting that decreasing hypothalamic MR expression prevents an increase in hypothalamic ENaC expression.

Dahl‐SS rats had dense *γ*ENaC immunoreactivity in dendrites of MNCs in the SON, whereas SD rats rarely had the immunoreactivities in dendrites. Because ENaC mediates a Na^+^ leak current that regulates the basal membrane potential of MNCs (Sharma et al. 2017), abnormal expression of ENaC in dendrites may result in abnormally depolarized local membrane potential that enhance postsynaptic excitability within the dendrite by depolarizing the local membrane potential. Therefore, it can be speculated that the enhanced excitatory synaptic inputs may sway the pattern and frequency of firing activity that ultimately regulates the release of VP (Poulain and Wakerley 1982). This may be why elevated plasma VP levels were observed in Dahl‐SS rats (Wainford and Kapusta 2010). Further studies need to be conducted to determine if Dahl‐SS rats have increased basal ENaC activity compared to normotensive rats.

The findings from this study suggest that hypothalamic ENaC expression, specifically the *γ*ENaC subunit, is involved in the etiology of salt‐sensitive hypertension development in Dahl‐SS rats. Given the role ENaC has in the resting membrane potential of VP neurons (Teruyama et al. 2012; Sharma et al. 2017) and in the enhanced ENaC activity in these neurons during high dietary salt consumption (Sharma et al. 2017), it is possible that VP neurons exhibit enhanced neuronal activity and subsequent hormone release that have large systemic effects.

## Conflict of Interest

No conflicts of interest, financial or otherwise, are declared by the authors.
